# The State of Immunity in Pregnancies Complicated By Intrauterine Infection of The Fetus

**DOI:** 10.34763/devperiodmed.20172104.384389

**Published:** 2018-01-02

**Authors:** Nikolay A. Shcherbina, Liudmyla A. Vygivska

**Affiliations:** 1 Department of Obstetrics and Gynecology №1, Kharkiv, Ukraine; 2 Department of Obstetrics, Gynecology and Pediatric Gynecology Kharkiv National Medical University Kharkiv, Ukraine

**Keywords:** urogenital infection, intrauterine infection, state of immunity

## Abstract

**Objectives:**

To study the state of immunity in pregnancies associated with urogenital infection and complicated by intrauterine infection.

**Material and methods:**

The comparative study involved the examination of 250 pregnant women with urogenital infection and ultrasonographic signs of intrauterine infection and their newborns in order to assess the state of cellular and humoral immunity components and nonspecific resistance. A direct prospective examination of pregnant women was carried out in the 2^nd^ and 3^rd^ trimesters of gestation. Depending on the outcome of each pregnancy on the basis of the follow-up of newborns, performed on the first day after birth, the patients were retrospectively divided into two groups. The study group included 93 (37.2%) pregnant women who developed intrauterine infection. The comparison group (n=157 (62.8%)) comprised pregnant-carriers of perinatally significant infection who gave birth to conditionally healthy children. The control group consisted of 50 healthy women with a physiological pregnancy.

**Results:**

In the gestation period under investigation, the development of intrauterine infection in pregnant women with urogenital infections was found to be associated with a deficiency of T-helpers / inducers, an increase in thymus-dependent lymphocyte killer activity, a high content of IL- 1β, TNF-α in the systemic circulation, and a decrease in the level of IL- 10 secondary to the oppression of the effector link of phagocytic neutrophils of peripheral blood.

**Conclusions:**

An increased concentration of systemic proinflammatory cytokines IL-1β, IL-6 and TNFα with a simultaneous decrease in the IL-10 content and suppression of the killing activity of peripheral blood phagocytes reflects the presence of an active inflammatory process in the mother-placenta-fetus system and can be one of the factors affecting the development of intrauterine infection in pregnancy, complicated by urogenital infection.

## Introduction

An increase in the incidence of intrauterine infection (IUI) is the most urgent problem for obstetricians, as it is one of the leading causes of morbidity and mortality in peri- and neonatal periods of fetal and neonatal development [1, 2].The incidence of IUI development in pregnancy, complicated by bacterial, viral or other infections, is 55.4-60.0% [3].

In Ukraine the frequency of intrauterine infections (IUI) ranges from 6 to 53%, reaching 70% among preterm infants. In the structure of perinatal mortality, the proportion of IUI ranges from 2 to 65.6% [4, 5, 6, 7].

It is known that intrauterine infection as a result of infectious matter invasion into the fetus does not always develop into a fetal infection, i.e. one in which the introduction of an infection by means of the penetration of a pathogen from an infected mother is expressed by a number of clinical manifestations in the early neonatal period [8].The latter is determined by the immune processes in the body of the pregnant woman, ensuring its physiological course.The development of intrauterine infection is associated with the fact that nonspecific functional “transient” immunosuppression accompanying pregnancy and providing control over child bearing without immune conflict, which is a systemic manifestation of the reactivity of the female body, contributes to an increase in its sensitivity to the e&ects of pathogenic factors, as a result of which immune mechanisms can become the reasons leading to the implementation of intrauterine infection a&ecting the development of the fetus and newborn [9, 10, 11, 12, 13].

Numerous studies have shown that infections are detected in almost every second birth [14, 15, 16]. In no small measure is this due to the fact that pregnancy can activate the persistence of latent infections.

The leading role in the pathogenesis of pathological conditions developing in the perinatal period is played by sexually transmitted infections [17, 18, 19]. Urogenital infections are the most common localization of the infectious matter in the human body, and during pregnancy their presence is associated with an increased risk of maternal and neonatal morbidity and mortality, even when the infection is asymptomatic [20, 21]. Such widespread prevalence of chronic urogenital diseases of viral, bacterial or mixed etiology can lead to an increase in the frequency of intrauterine infections, which, according to [22], leads to a disruption of postnatal adaptation of newborns and an increase in the number of infectious complications. At the same time transition of intrauterine infection into the infectious process in newborns depends on its stage and nature, on the state of immunity, which in such cases is characterized by a low level of both specific and nonspecific factors [23].

Thus, changes in the immune system during gestation may be due to an increased risk of infection.Therefore, it is relevant to study the issues related to the search for markers for assessing the risk of intrauterine infection and its implementation [24].

**The purpose of the study**: to assess the features of the state of immunity in pregnancies associated with urogenital infection and complicated by intrauterine infection.

## Material and methods

The study involved 250 pregnant women with urogenital infectious pathology and the presence of reliable signs of intrauterine infection.The gestation period was 28-37 weeks and was determined by the comparison of clinical and medical history data and ultrasonic fetometry findings.

The inclusion criteria were as follows: echographic IUI signs, singleton progressive unstimulated pregnancy, patient’s informed consent for the use of biological material for scientific purposes. Exclusion criteria were: multiple pregnancy, pregnancy with rhesus-sensitization, severe somatic pathology and chronic diseases in the decompensation stage (diseases of liver, kidney and cardiovascular system with impairment of their function), previous stimulation of ovulation, IVF, chromosomal abnormalities and congenital malformations of the fetus.

The distribution of patients by groups was carried out on the basis of retrospective analysis of immunity indices under investigation during the gestational period and the results of the follow-up examination of newborns performed in the first 72 hours after birth.The study group included 93 mothers, whose newborns had clinical signs of intrauterine infection.The comparison group comprised 157 patients with perinatally significant urogenital infections of di&erent origin, who gave birth to conditionally healthy children without signs of infection developing.The control group consisted of 50 conditionally healthy pregnant women with a physiological pregnancy at a similar gestational age in the absence of bacterial and viral infection on the basis of serological and microbiological research methods, whose infants were born without deviations in physical development and functional status. All the pregnant women gave natural birth at term (39-41 weeks).

Infectious status was determined by etiological interpretation using polymerase chain reaction (PCR) and enzyme immunoassay (ELISA) to detect *Cl. trachomatis, Ureaplasma urealiticum, Mycoplasma hominis, Mycoplasma genitalium, Nisseria gonorrheae, T. vaginalis*, as well as herpes (HSV-1/2, 6) and cytomegaly (CMV) viruses.

The state of cellular immunity components was identified by the phenotypic characteristics of peripheral blood lymphocyte populations and subpopulations, activation markers (HLA-DR) and receptors for IL-1β, IL-6, IL-10, TNF-α cytokines.The subpopulation composition of lymphocytes was determined on a FACSCalibur (USA) flow cytometer (CellQuest Pro software) using standard protocols. TNF-α was detected in blood serum by enzyme immunoassay with the use of reagent kits manufactured by ZAO Vector-Best (Novosibirsk, Russia) according to the attached instructions; TNF-α concentration was expressed in pg / ml. IL1-β, IL-6, IL-10 in blood serum was determined by solid-phase enzyme-linked immunosorbent assay using sets produced by ZAO Vector-Best (Novosibirsk, Russia) according to the manufacturer’s instructions. Optical density of the solutions was recorded on a microplate reader, and the concentration was expressed in pg/ml.

The state of humoral immunity components was assessed by the content of the immunoglobulins of the main classes, particularly G, A, M and circulating immune complexes in blood serum.The concentration of G, A, M immunoglobulins in blood serum of the pregnant women examined was determined by simple radial immunodi&usion [25], based on the immunological phenomenon of precipitation.The level of circulating immune complexes (CIC) in the serum of peripheral blood of the pregnant woman was assessed by spectrophotometry [26], based on the change in the magnitude of light scattering due to precipitation of antigen-antibody complexes in a 3.5% solution of polyethylene glycol (PEG, molecular weight 6000D).

Nonspecific resistance of the body was determined by phagocytic activity of peripheral blood neutrophils by the method of completed phagocytosis [27] using opportunistic staphylococcus (C-52 strain).

The majority of the women studied, i.e. 150 (60%) out of 250 had mixed viral-bacterial associations (VBAs), while virus-only infections were detected in only 50 (20%) and bacterial-only infections in 50 (20%) of the pregnant women. All the patients examined were comparable in age, social status, past gynecological and extragenital diseases.

Statistical analysis was carried out with the help of the “STATISTICA 6.1” application software (Russified version). Statistical significance of the intergroup di&erences was checked by the nonparametric Mann-Whitney test (MWT).

## Results and discussion

The assessment of the results obtained (tab. I) showed that in our studies cellular immunity was characterized by a significant decrease in the CD3+/CD4+ cell (suppressor T-Lymphocytes) subpopulation of circulating thymus-dependent peripheral blood lymphocytes in the pregnant women of the study group compared to the control group and comparison group and a significant increase in the total CD3+ CD16+/CD56+ (NKtot).

**Table I:** Phenotypic characteristics of lymphocytes and concentration of cytokines in the peripheral blood of pregnant in subjected pts (Me; LQ-UQ).

Indices	Comparison group (n=93)	Study group (n= ..157.)	Control group (n=50)
Т-total lymphocytes – (CD3+) (%)	72.4 (65.8-77.2)	68.8 (58.2-77.0)	75.5 (70.5-78.4)
Т-helpers/inducers- (CD3+/CD4+) (%)	40.8 (35.9-50.8)	32.3* (30.3-38.7)	47.2 (39.4-53.8)
Т-supressors/cytotoxic (CD3+/CD8+ ) (%)	22.2 (18.3-33.0)	20.8 (16.5-33.6)	24.3 (23.5-32.5)
В –lymphocytes (СD19+) (%)	10.0 (7.7-14.7)	10.8 (7.0-13.0)	10.5 (9.7-13.5)
ТNK-lymphocytes **(**CD3+CD16+/CD56+) (%)	7.6* (4.0-11.0)	9.8* (6.7-12.1)	5.5 (4.5-6.3)
NK –lymphocytes (CD3- CD16+/CD56+) ( %)	3.9 (2.6-7.5)	4.3 (3.1-7.7)	4.0 (2.5-7.4)
Т-activated lymphocytes **(**CD3+ HLA-DR) (%)	3.9 (2.6-7.5)	4.3 (3.1-7.7)	4.0 (2.5-7.4)
IL-1β pg/ml	18.8(9.8-20.3)	31.6* (24.1-42.6)	6.2 (0-8.5)
IL-6, pg/ml	12.4* (9.3-16.3)	20.6*(17.2-24.1)	5.2 (0 - 8.2)
IL-10, pg/ml	10.8 (9.6-15.5)	4.2*(0.1-5.0)	15.5(12.5-20.5)
TNFα pg/ml	56.1 (52.2-61.3)	70.8*(61.6-72.7)	34.6(28.1-42.4)

Note: *a significant difference from the control group and the comparison group, p<0.05 (MWT)

As it is known, intrauterine infection develops secondary to a severe depression of cellular immunity, and is probably the main etiological factor in suppressing the activity of the T-system of lymphocytes, in particular T-helper cells [28]).The depression of T-helpers can be triggered by the direct selective impact of viruses, bacteria, protozoa, fungi and other infections or can serve as the initial background predisposing to fetal infection.

Elevated levels of lymphocytes with CD3+ CD16 / CD56+ (NK tot) phenotype in the group of women with IUI development indicated an increase in the killer activity of lymphoid cells.

It should be noted that the relative content of B-lymphocytes (CD19+) in pregnant women with the development of maternal and fetal infections did not significantly differ from the control indices (p>0.05).

Assessment of the state of the cytokine system in both the study group and the comparison group without the development of intrauterine infection showed a significant increase in pro-inflammatory cytokines IL-1β, IL-6 and TNFα.

IL-1β concentration in the study group was almost 5 times higher than in the control group and 3 times that in the comparison group. A high IL-1β level in the systemic blood flow of patients in the study group may indicate activation of phagocytosis and cytolysis [29].

IL-6 content in peripheral blood serum of the study group was 4 times higher than in the control group and almost 2-fold that in the comparison group. Expression of IL-6 in biological fluids is always accompanied by great variability and depends on many factors a&ecting the intrauterine state of the fetus, including mixed infection [30]. In addition, as a mediator of subacute and chronic inflammation, IL-6 stimulates neutrophils and macrophages to local production of proinflammatory cytokines [31, 32, 33].

TNF-α content was almost 3 times higher than in the control group and 2 times that in the comparison group. TNF-α is an important cytokine involved in the development of inflammatory response, stimulating the activity of natural killers and cytotoxic lymphocytes, contributing to the development of a response to various infectious agents [34].

The results of IL-10 content assessment revealed an almost 4-fold and 2-fold decrease in its concentration in the main group in relation to the comparison and control group, respectively. A reduction of the content of this anti-inflammatory cytokine in the development of intrauterine infection indicates an impairment of adaptation of immunoregulatory mechanisms in IUI development [35].

Immunoglobulins play an important role as mediators in the cascade development of immune response and can partially determine the effectiveness of the final, effector responses of cellular immunity in inactivating and eliminating bacterial, viral and fungal antigens.

Assessment of humoral immunity (Ig A, M, G) indices showed a significantly higher content of IgM, a primary immune response mediator, in the pregnant women of the study group, as compared to the control and comparison group, which may indicate the activation of antibody-dependent cellular cytotoxicity in an active course of infection (tab. II).

**Table II:** The state of humoral immunity and nonspecific resistance of the peripheral blood in subjected pts (Мe; LQ- UQ).

Indices	Comparison group (n = 93)	Study group (n = 157)	Control group (n = 50)
Ig M g/l	1.68 (1.36-2.00)	1.92*(1.44-2.40)	1.21 (0.98-1.44)
Ig G g/l	12.1 (9.74-14.46)	12.98 (11.10-14.86)	12.3(10.60-15.11)
Circulating immune complexes,conventional units of optical density(explain shortcuts)	79.0 (38.5-109.5)	105.5* (81.6-129.4)	65.0 (44.0-67.0)
Phagocytic number %	88.0 (82.0 92.0)	92.0 (90.0-94.0)	89.0 (84.0- 90.0)
Phagocytizing neutrophils, st.un	4.4 (3.3-4.6)	4.3 (4.2- 4.7)	3.9 (3.3-4.4)
Bactericidal activity of neutrophils, %	37.7 (35.4-38.8)	39.9 (39.0-40.9)	37.7 (36.4-38.9)
Index of phagocytosis completeness, st. un.>1.0	0.94 (0.85- 0.99)	0.84*(0.72-0.93)	1.15 (0.98-1.4)

Note: *significant difference from the control group and comparison group, p <0.05 (MWT)

IgA concentration in all the groups of patients (under investigation) was within the normal range, but it was in the upper limit of its reference values, which corresponded to the literature data on the increase of this index during the entire gestation period, reaching a maximum in the 2^nd^ and 3^rd^ trimesters [36]. IgG content in all the groups under investigation was within the normal range.

The content of circulating immune complexes (CIC) is an integral indicator of the functional activity of humoral immunity. The study showed that CIC concentration in the serum of the patients of the study group was significantly higher (p <0.05) than in the control group.

CIC formation is an obligatory component of normal immune response, one of the most important humoral mechanisms of antigen elimination and a universal defense mechanism aimed at maintaining the internal environment of the body. Furthermore, CIC is able to interact with most immunocompetent cells and cells of the monocyte-phagocytic system, which is expressed in the production and secretion of biologically active substances.

Neutrophils are the main effector cells controlling infectious agents. Assessment of the functional reserve of phagocytizing neutrophils of peripheral blood of the pregnant women under investigation showed that the absorption capacity of phagocytes in all the groups of the patients examined was practically on the same level as their bactericidal activity (BAN). However, the latter was accompanied by a significant (p<0.05) decrease in the digesting capacity of phagocytizing neutrophils in the blood of the pregnant women who developed intrauterine infection – the index of phagocytosis completeness was 0.89 st.un. against 1.15 and 0.94 st.un. in the control and comparison group, respectively (tab. II).

The physiological course of pregnancy, in the absence of an infectious factor, as a result of an increase in the total number of neutrophils in blood enhancing their ability to capture opsonized antigenic material, and increasing their bactericidal activity, is characterized by the development of a powerful and mobile neutrophil reserve to perform full phagocytic function, indicating that CIC content is under effective control, which is confirmed by our study. In the development of intrauterine infection, high CIC concentration in blood serum of the patients of the study group is associated with insufficiency of their clearance from the blood flow [37].

Thus, our analysis of the data obtained allows us to conclude that the state of immunity in pregnant women with urogenital infections and IUI development is characterized by a deficiency of T-helper/inducer lymphocytes, an increase in CD3+ CD16+ / CD56+ cells, an imbalance of anti- and pro-inflammatory cytokines, with a prevalence of the latter. The development of the inflammatory process in the mother-placenta-fetus system, which is confirmed by the high content of IL-1β and TNF-α in the systemic circulation, is accompanied by the activation of the humoral immune link, which is manifested by an increase in the level of circulating immune complexes. This fact may indicate that CIC accumulation in peripheral blood during the implementation of intrauterine fetal infection is not so much triggered by their excessive formation in response to antigenic stimulation, but rather by the lack of mechanisms for their elimination, which is confirmed by our findings on the decrease in the killing activity of the effector link of phagocytizing neutrophils of peripheral blood.
